# *Galkinius* Perreault, 2014 or *Darwiniella* (Anderson, 1992)? A new coral-associated barnacle sharing characteristics of these two genera in Pacific waters (Crustacea, Cirripedia, Thoracica, Pyrgomatidae)

**DOI:** 10.3897/zookeys.719.12471

**Published:** 2017-12-07

**Authors:** Benny Kwok Kan Chan, Jennie Chien Wen Liu

**Affiliations:** 1 Biodiversity Research Center, Academia Sinica, 128 section 2, Academia Road, Taipei 115, Taiwan; 2 Institute of Ecology and Evolutionary Biology, National Taiwan University. Taipei 106, Taiwan

**Keywords:** Barnacles, corals, Pyrgomatidae, host specificity

## Abstract

A new species of coral associated barnacle (Balanomorpha: Pyrgomatidae) sharing morphological features of *Darwiniella* (Anderson, 1992) and *Galkinius* Perreault, 2014 is described. It has a fused shell and opercular plates, characteristic of *Darwiniella*. However, the morphology of the tergum and somatic body are closer to *Galkinius*. Sequence divergence of mitochondrial DNA 12S rDNA and COI reveals this new species clusters with the *Galkinius* clade. Therefore this new form is assigned to the genus *Galkinius*, as *G. maculosus* **sp. n.** Concomitantly the diagnosis of *Galkinius* is emended to include species with fused or four- plated shells and fused opercular plates. The new species is distinct from all *Galkinius* species in having a fused shell. It inhabits the corals *Lobophyllia* spp. and is distributed from the Dongsha Atoll in the South China Sea, Orchid Island of Taiwan in the Pacific Ocean, to Madang in Papua New Guinea waters.

## Introduction

Barnacles in genus *Galkinius* Perreault, 2014 are coral associated species of the family Pyrgomatidae. Species of *Galkinius* were originally grouped under the genus *Creusia* Leach, 1817 by Darwin (1854). Ross and Newman (1973) revised the taxonomy of pyrgomatid barnacles and redefined *Creusia* as having a 4-plated shell but a fused scutum and tergum. Galkin (1986) established a new genus *Utinomia* Galkin, 1986 to accommodate *Creusia* species which had a broad adductor plate and a rostral tooth in the scutum. However, the generic name *Utinomia* is preoccupied by *Utinomia* Tomlinson, 1963 for an acrothoracican barnacle (Tomlinson 1963). Ross and Newman (1995) renamed *Utinomia* as *Galkinia*, and designated *G. indicum* (Annandale, 1924) as the type species. Perreault (2014) pointed out the generic name *Galkinia* Ross & Newman, 1995 was preoccupied by a genus of fossil fish, *Galkinia* Ghekker, 1948 (Actinopterygii: Pholidophoriformes). He therefore renamed *Galkinia* as *Galkinius* Perreault, 2014, thereby continuing to recognize Galkin’s contribution to cirripede taxonomy.

According to Ross and Newman (1973) and Ogawa (2000), there were three *Galkinius* species including *G. decima* (Ross & Newman, 1973), *G. indica* (Annandale, 1924), and *G. supraspinulosa* Ogawa, 2000. Chan et al. (2013) subsequently identified five new species of *Galkinius* in Taiwan waters (also see Tsang et al. 2014). Simon-Blecher et al. 2016 revealed there is geographical variation in the opercular plate morphology of *Galkinius* in the Indo-Pacific waters, and that there were four additional un-named cryptic species in the region suggesting there was considerably more diversity to be explored in the Pacific.

In this study, 39 specimens of a new pyrgomatid barnacle were collected in the Pacific region (Dongsha Atoll, Orchid Island in Taiwan waters and Madang in Papua New Guinea). This undescribed species has four plated shells and a fused operculum plate, which are characteristics of *Darwiniella* (Anderson, 1992). However, the somatic body and the shape of tergum is very similar to *Galkinius*. From sequence divergence in mitochondrial 12S rDNA (12S) and cytochrome c oxidase subunit I (COI) gene, this new species is closer to *Galkinius* than it is to *Darwiniella*. Therefore it was decided to classify it in the genus *Galkinius*. The diagnosis of *Galkinius* is emended to accommodate this new species of *Galkinius* which shares many characters with *Darwiniella*.

## Materials and methods

### Specimen sampling and morphological analysis

**Figure 1.**
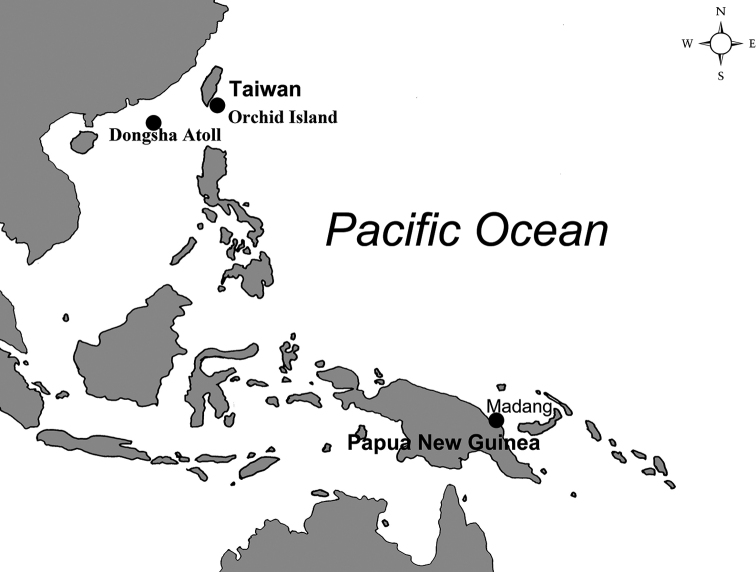
Collection sites of the *Galkinius maculosus* sp. n. in the Pacific waters.

The undescribed *Galkinius* species was sampled in Pacific waters, including the outlying islands of Taiwan waters (Dongsha Atoll in the South China Sea, Orchid Island in the Pacific Ocean) and Madang in the waters of Papua New Guinea (Fig. 1). Barnacles were collected with small pieces of their coral host using hammers and chisels when SCUBA diving and then fixed in 95% EtOH. Holotype and paratype specimens are stored in the Biodiversity Museum of the Academia Sinica, Taipei, Taiwan (**ASIZCR**), and the National Museum of Natural History, Paris, France (**NMNH**). Additional specimens are stored in the Coastal Ecology Laboratory, Academia Sinica, Taiwan (**CEL**). After barnacle specimens were removed from the host coral with forceps, they were examined under light microscopes (LM; Zeiss Scope A1) and scanning electron microscopes (SEM; FEI Quanta 200) to further describe their morphological characters, including hard parts (shell and opercular valves) and the somatic body (cirri, penis and mouth parts). To determine the structure and articulations between individual shell parts, all the barnacle tissue, coral tissue and other organic debris adhering to the shell and the opercular valves were carefully removed by forceps, and then 1.5% bleach was used to digest the remaining tissue. After immersion in bleach for approximately three hours, the remaining organic tissue could then be torn off easily by forceps. The cleaned shells were rinsed with water for approximately 30 minutes and air-dried. The shell and opercular valves were coated with gold and then observed under SEM following the methods of Chan et al. (2013). The somatic body, including the six pairs of cirri, the penis, and the mouth parts were dissected out and observed under LM. Setal descriptions are based on Chan et al (2008).

### Molecular analysis

Total genomic DNA was extracted from soft tissue of individual specimens using a Qiagen (Chatsworth, CA) QIAquick Tissue Kit following the manufacturer’s instructions. Partial sequences of mitochondrial genes 12S rDNA (12S) and cytochrome c oxidase subunit I (COI) were amplified by polymerase chain reaction (PCR) with primer 12S-FB and 12S-R2 (Tsang et al. 2009), and COI-F5 5’ AAACCTATAGCCTTCAAAGCT 3’ and COI-R4 5’ GTATCHACRTCYATWCCTACHG 3’, respectively. The PCR solution contained 40 ng of template DNA, 5 μl Taq DNA Polymerase Master Mix (1.5 mM MgCl_2_; Ampliqon, Denmark), 1 μM of each primer, and ddH_2_O with a final volume of 10 μl. The PCR reaction was conducted under the following conditions: 2 min at 95 °C for initial denaturing, 35 cycles of 30 sec at 95 °C, 1 min at 48 °C, 1 min at 72 °C with a final extension for 5 min at 72 °C. The PCR products were then purified using the DNA Gel purification kit (Tri-I Biotech, Taipei, Taiwan). Direct sequencing of the purified PCR products was performed on an ABI 3730XL Genetic Analyzer with BigDye terminator cycle sequencing reagents (Applied Biosystems, Foster City, California, USA). Sequences were then aligned with BioEdit Sequence Alignment Editor V7.2.5 (Hall et al. 2013) using default settings and adjusted by eye.

The genealogical relationships of specimens based on 12S were inferred using both Maximum Composite Likelihood model, 1000-replicate Neighbor-Joining (NJ) method and T92 model, 1000-replicate Maximum Likelihood (ML) method implemented in MEGA v7.0.14 (Kumar et al. 2016). We reconstructed the relationship between three species of *Darwiniella* (*Darwiniella angularis*, *D. conjugatum*, and *D. maculosus* sp. n.) and eight *Galkinius* Perreault, 2014 species (*Galkinius adamanteus* Chan, Chen & Lin, 2013, *G. equus* Chan, Chen & Lin, 2013, *G. decima* (Ross & Newman, 1973), *G. tabulatus* Chan, Chen & Lin, 2013, *G. depressa* Chan, Chen & Lin, 2013, *G. altiapiculus* Chan, Chen & Lin, 2013, *G. trimegadonta* Chan, Chen & Lin, 2013, and *G. indica* (Annandale, 1924). Additionally, five specimens of the coral barnacle *Nobia grandis* Sowerby, 1839 were used as the outgroup. Additionally, three sequences of *Darwiniella* spp. and four sequences of *Galkinius* species form Malay and Michonneau 2014 were downloaded from EMBL and added into the analysis. The evolutionary distance (number of base differences per site) between sequence pairs was calculated with uncorrected p-distance and Tamura 3-parameter model (T92) models by MEGA.

## Results

### Systematics

#### Suborder Balanomorpha Pilsbry, 1916

##### Family Pyrgomatidae Gray, 1825

###### Subfamily Pyrgomatinae Gray, 1825

####### Genus Galkinius

Taxon classificationAnimaliaSessiliaPyrgomatidae

Perreault, 2014

######## Diagnosis (emended).

Shell wall fused or four plated, flat, with high radial ridges at the junction with coral skeleton. Scutum and tergum fused, the two parts being approximately subequal. Adductor ridge and lateral depressor muscle scars absent, adductor plate and rostral tooth present. Tergal spur well developed and wide. Apertural frill coloured and spotted. Maxilla and cirri with numerous dark spots and bands.

######## Type species.

*Galkinius indica* (Annandale, 1924).

######## Remarks.

In the original diagnosis of *Galkinius*, the shell consisted of four separated plates and the fused scutum and tergum, which differs from *Darwiniella* which has a fused shell as well as a fused scutum and tergum. In the present study, a new species of *Galkinius* was identified as having a fused shell wall. Therefore it is necessary to emend the diagnosis of *Galkinius*to accommodate this species (see discussion below). *Galkinius* differs from *Darwiniella* in having much wider tergal spur and tergal furrow. Height of the adductor ridge of the scutum in *Darwiniella* is much greater than in species of *Galkinius*. In *Darwiniella*, the height of adductor ridge is approximately 2/3 to 1/2 total height of scutum. In *Galkinius*, height of adductor plate is often approximately 1/3 of the total height of scutum. Maxilla of *Galkinius* and cirri with large number of coloured spots and bands, when compared to *Darwiniella*. The apertural frills of *Darwniella angularis* and *D. conjugatum* are white, while *Galkinius* has a coloured or spotted aperture frill.

####### Galkinius maculosus sp. n.

Taxon classificationAnimaliaSessiliaPyrgomatidae

http://zoobank.org/E4DA73E3-3E73-4F6C-B238-704943136D65

######## Material examined.

HOLOTYPE. ASIZCR000343, SE of Dongsha outer atoll, Taiwan (20°36.937'N, 116°53.143'E), June 2015, coll. Pei-Chen Tsai,Yao-Fong Tsao, and Yen-Wei Chang, on coral host *Lobophyllia* de Blainville, 1830 sp. PARATYPES. ASIZCR000344, NW of Dongsha Atoll, Taiwan (20°36.173'N, 116°52.110'E), May 2015, coll. Pei-Chen Tsai,Yao-Fong Tsao, and Yen-Wei Chang, on coral host *Lobophyllia* sp. ASIZCR000345, NE of Dongsha Atoll, Taiwan (20°46.616'N, 116°47.203'E), May 2015, coll. Pei-Chen Tsai,Yao-Fong Tsao, and Yen-Wei Chang, on coral host *Lobophyllia* sp., ASIZCR000346, Dongsha wreck (20°42.282'N, 116°42.097'E), May 2014, coll. Chen Hsi-Nien, and Pei-Chen Tsai, on coral host *Lobophyllia agaricia* (Milne Edwards & Haime, 1849). MNHN-IU-2016-8720, PKK2, Madang, Papua New Guinea, November 2012, coll. B.K.K. Chan, on coral host *Lobophyllia radians* (Milne Edwards & Haime, 1849) Edwards & Haime, 1849. ADDITIONAL SPECIMENS. CEL-LAN-075-09, Rock Yunuyen, Orchid Island, Taiwan (22°08.111'N, 121°52.000'E), October 2007, coll. B.K.K. Chan, coral host unknown. CEL-DSA-012-1-9, Dongsha wreck, Taiwan (20°42.282'N, 116°42.097'E), May 2014, coll. Pei-Chen Tsai, on coral host *Lobophyllia agaricia*. CEL-DSA-075, Dongsha wreck, Taiwan (20°46.767'N, 116°48.402'E), August 2015, coll. Pei-Chen Tsai, Yao-Fong Tsao, and Yen-Wei Chang, on coral host *Lobophyllia* sp., CEL-DSA-084-1, 2, 4, 5, data same as paratype ASIZCR000344. CEL-DSA-097-1, 2, data same as paratype ASIZCR000345. CEL-DSA-117-1-5, data same as holotype. CEL-DSA-131-3, Dongsha wreck, Taiwan (20°42.380'N, 116°42.088'E), May 2015, coll. Pei-Chen Tsai, on coral host *Lobophyllia* sp., CEL-DSA-201, SE of Dongsha outer atoll, Taiwan (20°36.825'N, 116°53.012'E), May 2016, coll. Pei-Chen Tsai, Yao-Fong Tsao, and Yen-Wei Chang, on coral host *Lobophyllia* sp., PNG-020-01, 02, data same as paratype MNHN-IU-2016-8720.

######## Diagnosis.

*Galkinius* with fused shell wall, spotted aperture frill; cirri, maxilla, and penis with dark spots, scutum with relatively narrow adductor plate, tergum with wide spur.

######## Description.

**Figure 2.**
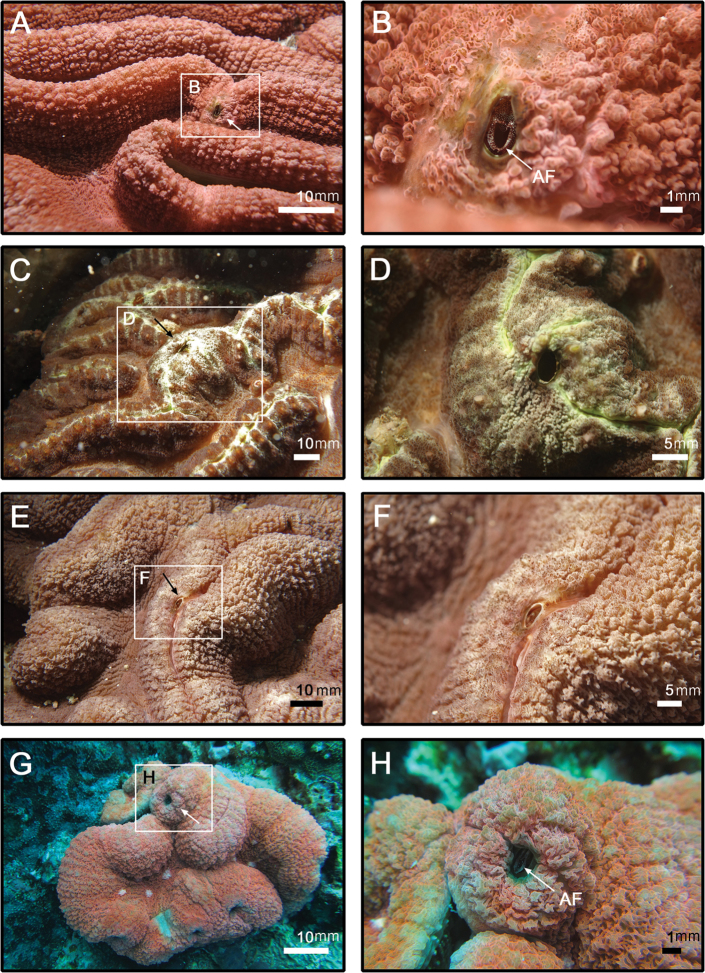
*In-situ* underwater photo of *Galkinius maculosus* sp. n. **A** Additional specimen CEL-DSA-117 (white arrow), on coral *Lobophyllia* sp., NE of Dongsha Atoll, Taiwan **B** Magnified photo of the barnacle (CEL-DSA-117) showing the spotted aperture frill **C** Additional specimen CEL-DSA-075 (white arrow), on coral *Lobophyllia* sp., SE of Dongsha Atoll, Taiwan **D** Magnified photo of the barnacle (CEL-DSA-075) **E** Additional specimen CEL-DSA-097 (white arrow), on coral *Lobophyllia* sp., NE of Dongsha Atoll, Taiwan **F** Magnified photo of the barnacle (CEL-DSA-097) **G** Additional specimen CEL-DSA-201 (white arrow), on coral *Lobophyllia* sp., Northeast of Dongsha Atoll, Taiwan **H** Magnified photo of the barnacle (CEL-DSA-201) showing spotted aperture frill. (AF: aperture frill).

**Figure 3.**
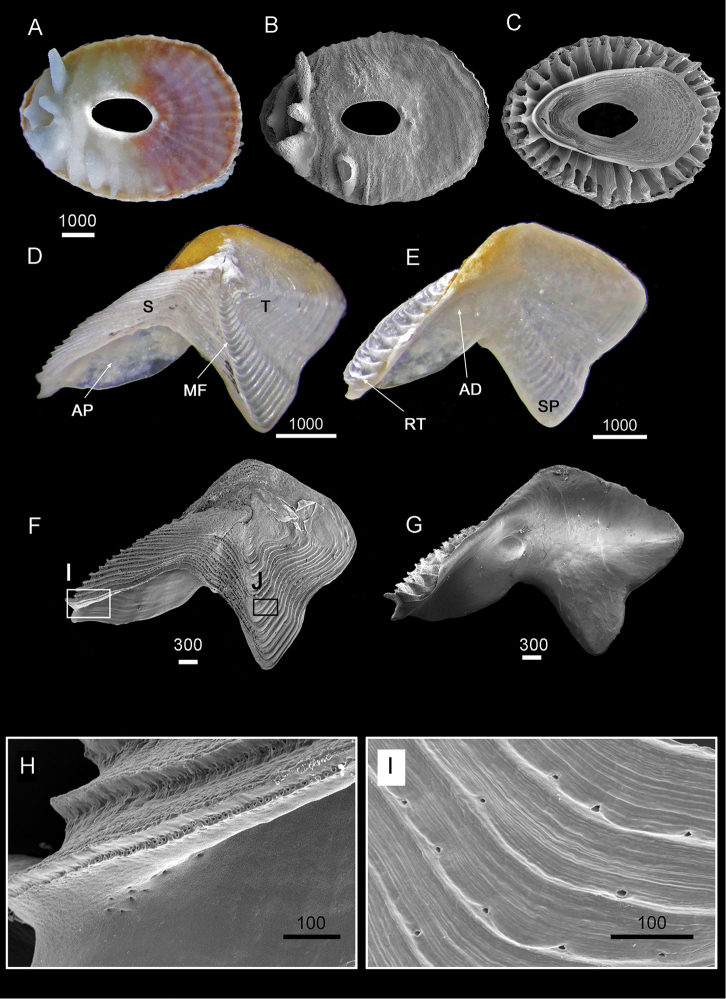
Shell and opercular plates of *Galkinius maculosus* sp. n. **A** (LM) and **B** (SEM) of dorsal view of fused shell (Holotype, ASIZCR000343) **C** Ventral view of shell (SEM) with internal rids radiating from rim of inner operculum (CEL-LAN-075-09) **D** Dorsal view and **E** Ventral view of fused scutum and tergum (ASIZCR000343) **F** Dorsal view (CEL-DSA-012-9) and **G** Ventral view (CEL-DSA-012-9) of fused scutum and tergum under Scanning Electron microscope **H** Horizontal striations on external surface of scutum **I** Horizontal striations on external surface of tergum. Scale bars in µm. Abbreviations: AP: adductor plate, S: scutum, T: tergum, MF: medial spur furrow, RT: rostral tooth, AD: adductor muscle scar, SP: spur.

(Description based on holotype: basal diameter 12 mm, rostro-carinal orifice diameter 9 mm). From i*n-situ* observation, shell of barnacles covered by thick coral tissue, aperture frill black with white spots (Fig. 2), colouration did not changing after preservation in 95% EtOH. Shell oval, plates fully fused, pink externally after bleach treatment, external surface smooth (Fig. 3A, B). Base of shell with 30–40 internal rids radiating from rim of inner operculum (Fig. 3C). Orifice oval, long, narrow, about 1/3 length of rostro-carinal diameter.

Scutum and tergum white, plates fused without junctions (Fig. 3D–G). Width of scutum similar to width of tergum. Scutum triangular, transversely elongated, width two times longer than height. Occludent margin straight, with 6–8 rostral teeth basally along ventral surface of occludent margin, teeth gradually increasing in size from apex to base (Fig. 3D–G). Ventral view with oval-shaped adductor muscle scar. Dorsal view with horizontal striations, each bearing rows of small pores (Fig. 3H). Adductor plate convex, extending below basal margin half height of scutum (Fig. 3D, F). Tergum trapezoid, three times higher than scutum. Tergum apex pronounced, lateral depressor muscle crests not apparent. Spur wide, reaching one third width of basal margin of tergum, base convex, height of scutal side of spur three times longer than carinal side, height of spur about one third height of tergum. Dorsal surface with middle spur furrow, curving slightly from the basal margin towards carinal margin (Fig. 3D). Dorsal surface with horizontal striations, each bearing rows of small pores (Fig. 3I).

**Figure 4.**
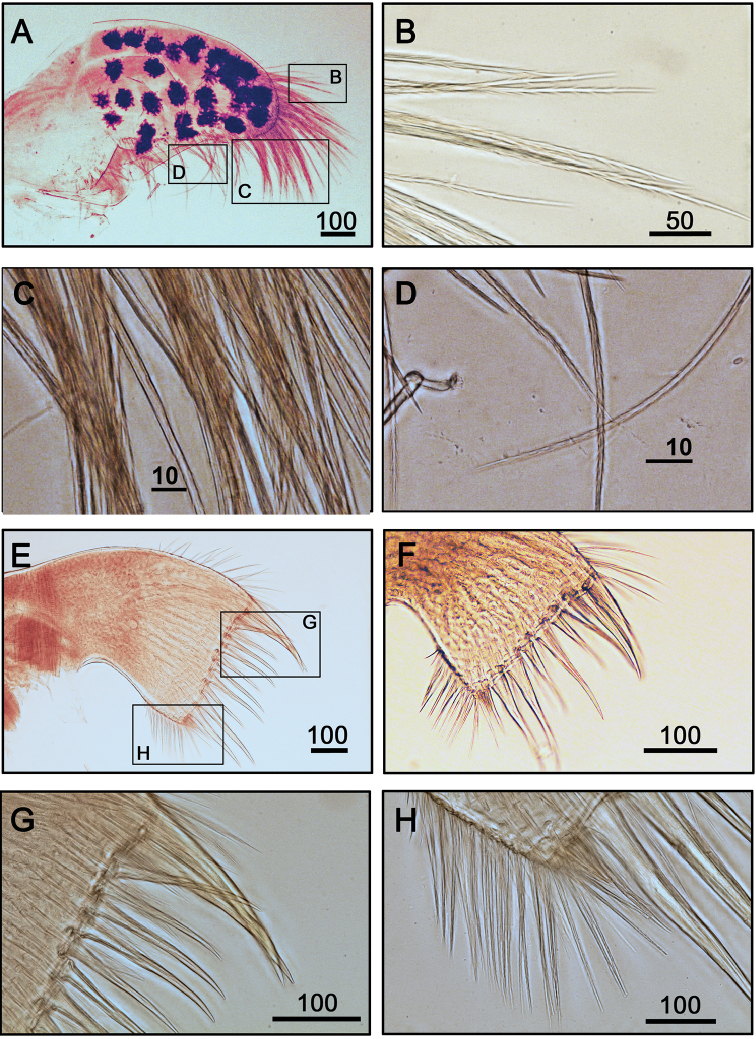
Maxilla and maxillule of *Galkinius maculosus* sp. n. **A** Maxilla oval, with dark spots (ASIZCR000343) **B–D** Serrated setae on margin **E** Maxillule (ASIZCR000343) **F** Maxillule (CEL-DSA-012-6) **G** Large simple setae on straight cutting edge **H** Simple setae on lateral margin. Scale bars in µm.

**Figure 5.**
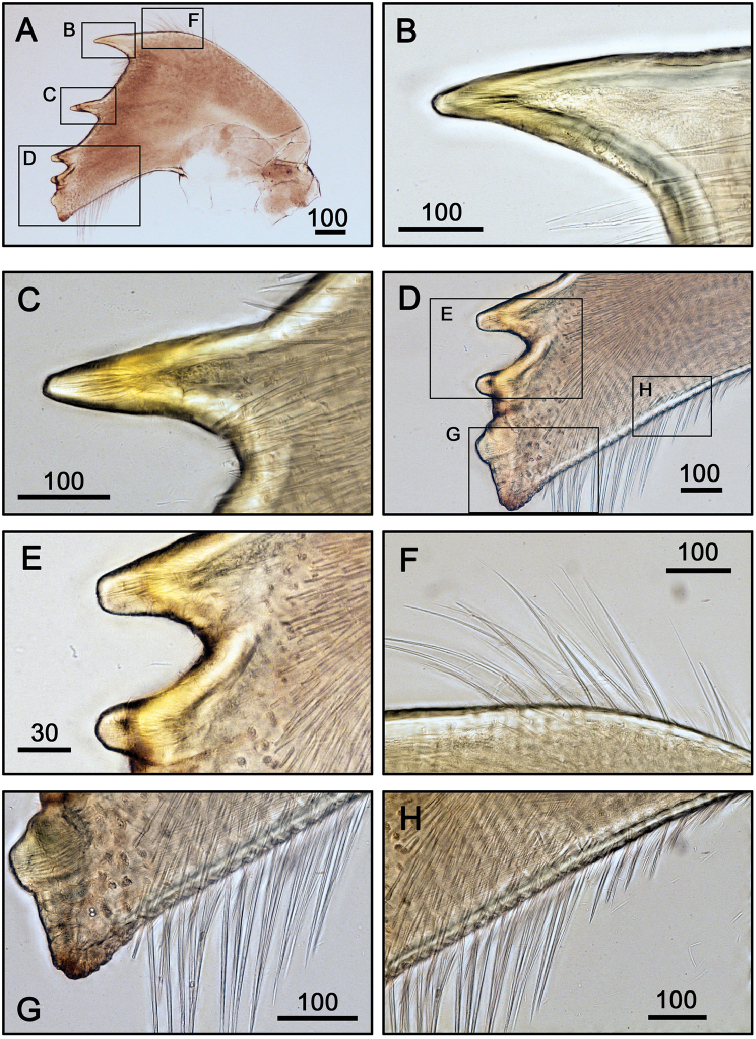
Mandible of *Galkinius maculosus* sp. n. **A** Mandible (ASIZCR000343) **B** First teeth of mandible **C** Bidentate second tooth **D** Lower margin and inferior angle with simple setae **E** Bidentate third and fourth teeth **F** simple setae on lateral margin **G** Inferior angle with simple setae **H** Lower margin with simple setae. Scale bars in µm.

**Figure 6.**
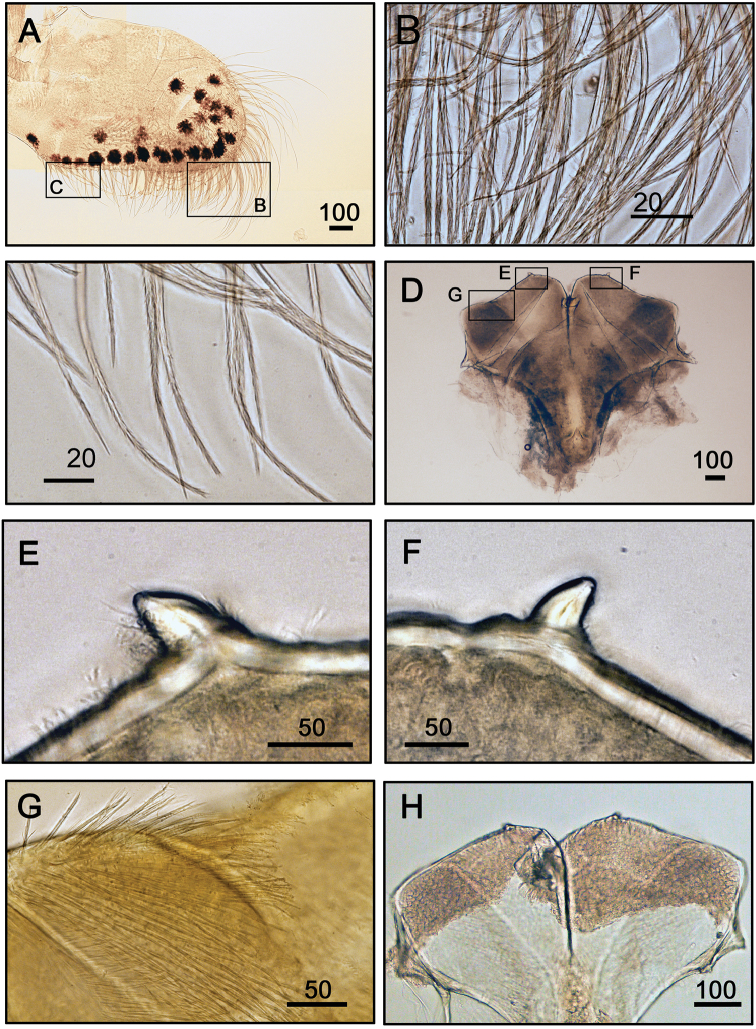
Mandibular palp and labrum of *Galkinius maculosus* sp. n. **A** Mandibular palp showing black spots (ASIZCR000343) **B** Serrulate setae distally **C** Serrulate setae on interior margin **D** Bilobed labrum with V-shaped notch between two lobes (ASIZCR000343) **E** Tooth on labrum **F** Tooth on labrum **G** Surface of labrum with simple setae **H** Bilobed labrum (CEL-DSA-012-6). Scale bars in µm.

Maxilla oval, with dark spots (Fig. 4A), serrulate setae distally (Fig. 4B, C) and along inferior margin (Fig. 4D). Maxillule cutting edge straight without notch, bearing row of 9–12 large setae (inconsistent, withtwo specimens with 12 and 9 large setae, Fig. 4E, F, respectively). Region close to cutting edge with fine simple setae (Fig. 4H). Mandible with four teeth (Fig. 5A). First teeth largest and sharp (Fig. 5C). Second, third, and fourth teeth bidentate (Fig. 5C, E). First and second teeth well separated than remainder, third to fifth teeth smaller than first and second teeth. First three teeth occupying 3/4 length of cutting edge. Lower margin short, about 1/16 length of total length of mandible. Lateral side and lower margin of mandible bearing simple setae (Fig. 5D–H). Mandibular palp rectangular, elongated (Fig. 6A), bearing serrated setae distally (Fig. 6B) and along interior margin (Fig. 6C). Labrum bilobed, V-shaped notch between two lobes, one sharp tooth on each side of notch (Fig. 6D–G) (consistent in two specimens, Fig. 6D, H).

**Figure 7.**
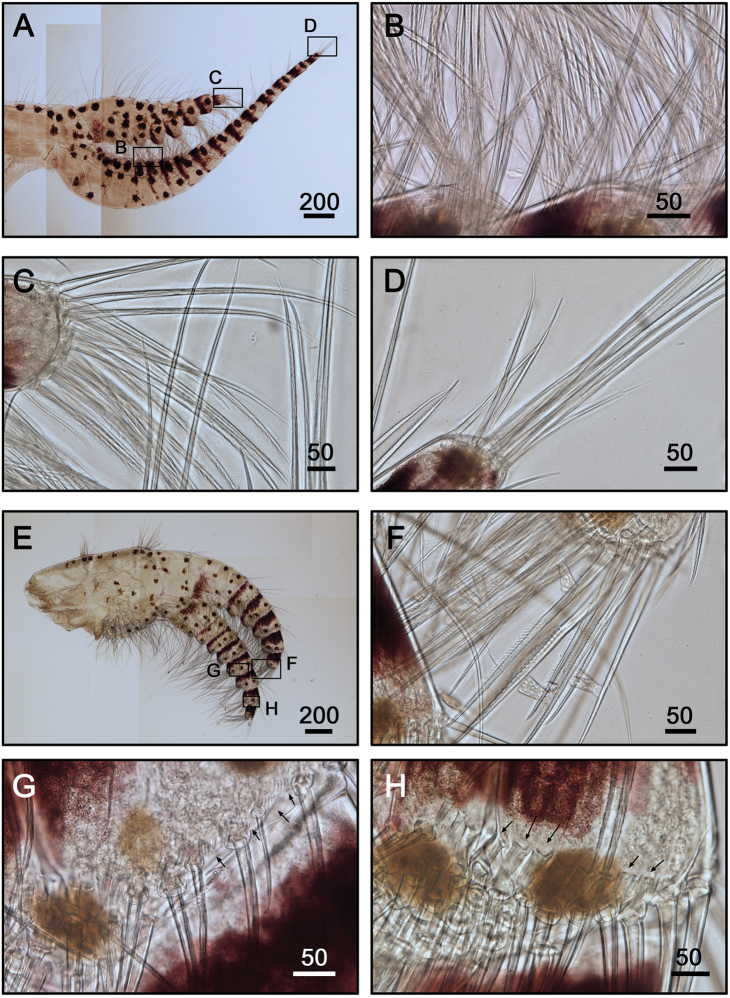
Cirrus I, II of *Galkinius maculosus* sp. n. **A** Cirrus I with dark spots, posterior ramus shorter than anterior one (ASIZCR000343) **B** Serrulate setae on anterior ramus **C** Simple and serrulate setae on the distal segment of posterior ramus **D** Simple and bidentate serrulate setae on the distal segment of anterior ramus **E** Cirrus II with dark spots and stripes on each segment, rami almost equal length (ASIZCR000343) **F** Simple and bidentate serrulate setae on the distal segment of anterior ramus **G** Fan-shaped denticles at the margins of middle segment (indicated by arrows) **H** Series of conical spines at the margin of distal segments (indicated by arrows). Scale bars in µm.

**Figure 8.**
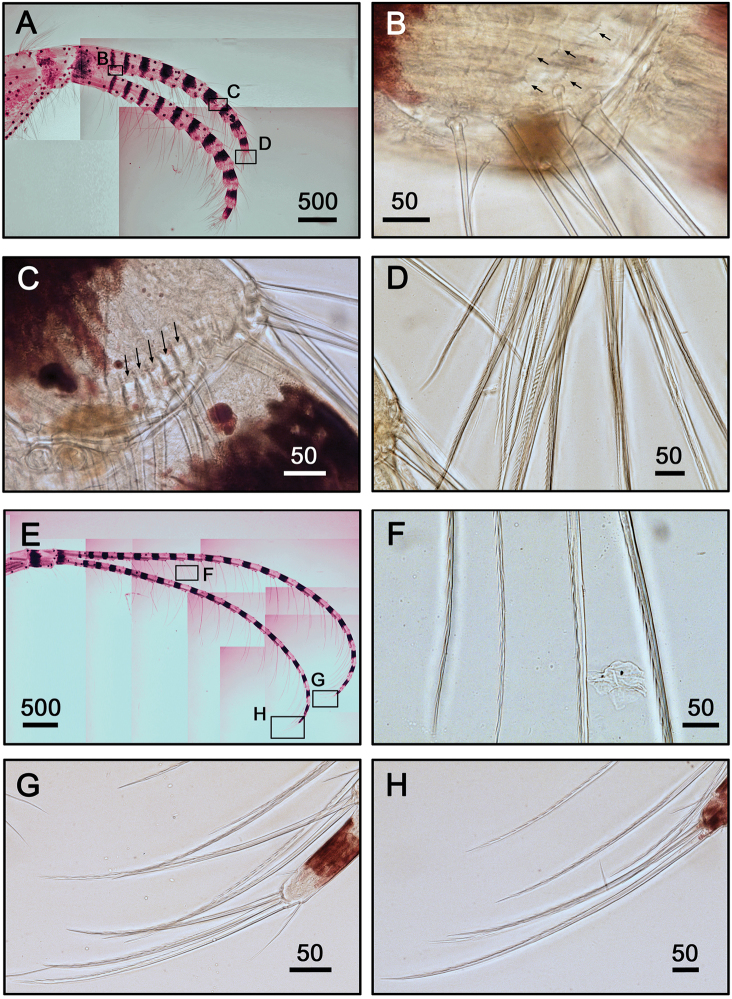
Cirrus III, IV of *Galkinius maculosus* sp. n. **A** Cirrus III with dark spots and stripes on each segment, rami almost equal length (ASIZCR000343) **B** Fan-shaped denticles on the surface of basal segments of posterior ramus (indicated by arrows) **C** Series of conical spines at the margin of distal segments of posterior ramus (indicated by arrows) **D** Simple and serrulate setae on the distal segment of posterior ramus **E** Cirrus IV, with stripes on each segment, rami almost equal length (ASIZCR000343) **F** simple and serrulate setae on intermediate segment **G** Simple and serrulate setae on the distal segment of posterior ramus **H** Simple and serrulate setae on the distal segment of anterior ramus. Scale bars in µm.

**Figure 9.**
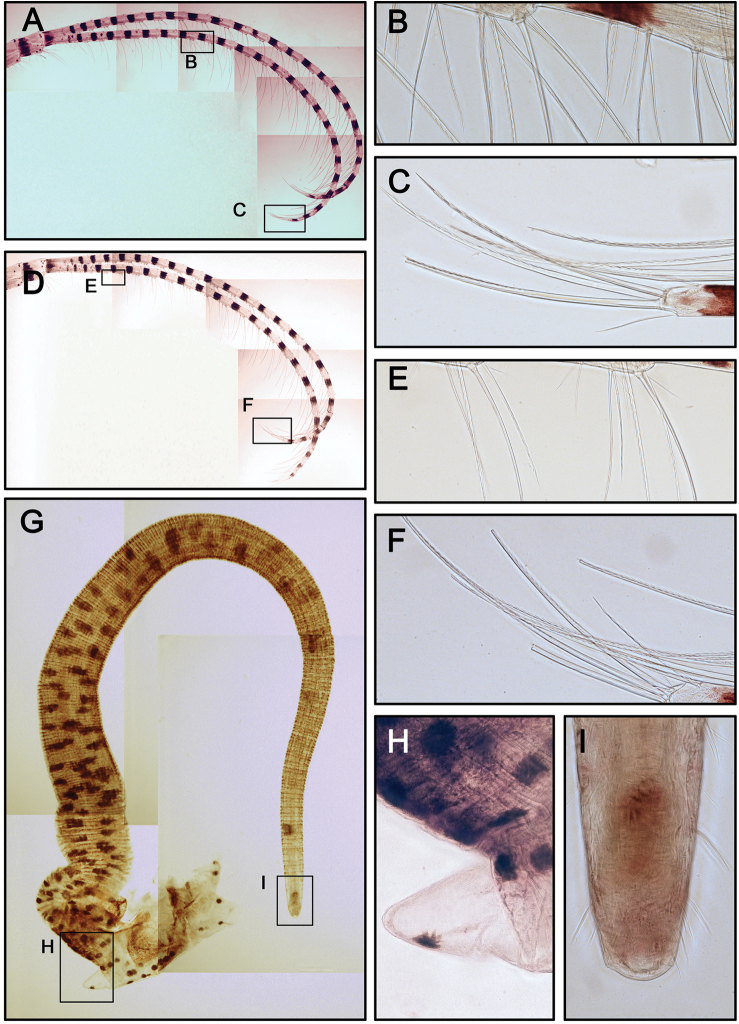
Cirrus V, VI and penis of *Galkinius maculosus* sp. n. **A** Cirrus V, with stripes on each segment, rami almost equal length (ASIZCR000343) **B** Intermediate segment with 4 pairs of serrulate setae **C** Serrulate setae on the distal segment of anterior ramus **D** Cirrus VI, with stripes on each segment, rami almost equal length (ASIZCR000343) **E** Intermediate segment with 4 pairs of serrulate seta **F** Serrulate setae on the distal segment of posterior ramus **G** Penis with dark spots (ASIZCR000343) **H** Basi-dorsal point of penis **I** Apex of penis with short simple setae. Scale bars in µm.

Cirrus I with rami unequal. Dark spots and stripes on each segment of anterior and posterior rami (Fig. 7A). Posterior ramus short (nine segments), bearing serrate setae (Fig. 7B), the anterior edges of the rami carry simple and serrulate setae (Fig. 7C). Anterior ramus long (17 segments), slender, anterior edges of the segments bearing simple and bidentate serrulate setae (Fig. 7D). Cirrus II rami sub-equal. Dark spots and stripes on each segment of anterior and posterior rami (Fig. 7E) Anterior ramus (nine segments) and posterior ramus (seven segments), bearing serrulate setae. Anterior edges of both anterior and posterior rami with both simple and bidentate serrulate setae (Fig. 7F). Fan-shaped denticles present at the margins of middle segments (Fig. 7G) and conical spines present at the margin of distal two to three segments (Fig. 7H). Cirrus III rami subequal (Fig. 8A), dark spots and stripes exist on each segment of anterior and posterior rami. Anterior ramus (12 segments) and posterior ramus (10 segments), with simple and serrulate setae. Fan-shaped denticles (Fig. 8B) present at the surface of basal segments of posterior ramus Conical spines present at the margin of the distal three up to eight segments at both anterior and posterior rami (Fig. 8C). Anterior sides of both anterior and posterior rami with bidentate serrulate setae (Fig. 8D). Cirrus IV-VI long, slender, with equal rami length. Number of segments on Cirrus IV (22, 20) (Fig. 8E), Cirrus V (24, 24) (Fig. 9A), Cirrus VI (23, 23) (Fig. 9D). Stripes exist on each segment of the ramus (Figs 8E, 9A, 9D). Intermediate segments of Cirrus IV-VI has four pairs of serrulate setae (Figs 8F, 9B, C, E, F), distal pair longest, proximal pair shortest. Penis long (about one and a half times length of Cirrus VI), annulated, with scattered irregular dark spots (Fig. 9G). Pedicel with basidorsal point (Fig. 9G, H), apex of penis with short, simple setae (Fig. 9I).

######## Etymology.

The name *maculosus* means dappled or mottled, and therefore denotes the spots scattered around the aperture frill, maxilla, palp, Cirrus I-VI, and penis of this species.

######## Distribution.

Taiwan waters (Dongsha Atoll in the South China Sea, Orchid Island in the Pacific Ocean), Madang, Papua New Guinea.

## Molecular analysis

**Figure 10.**
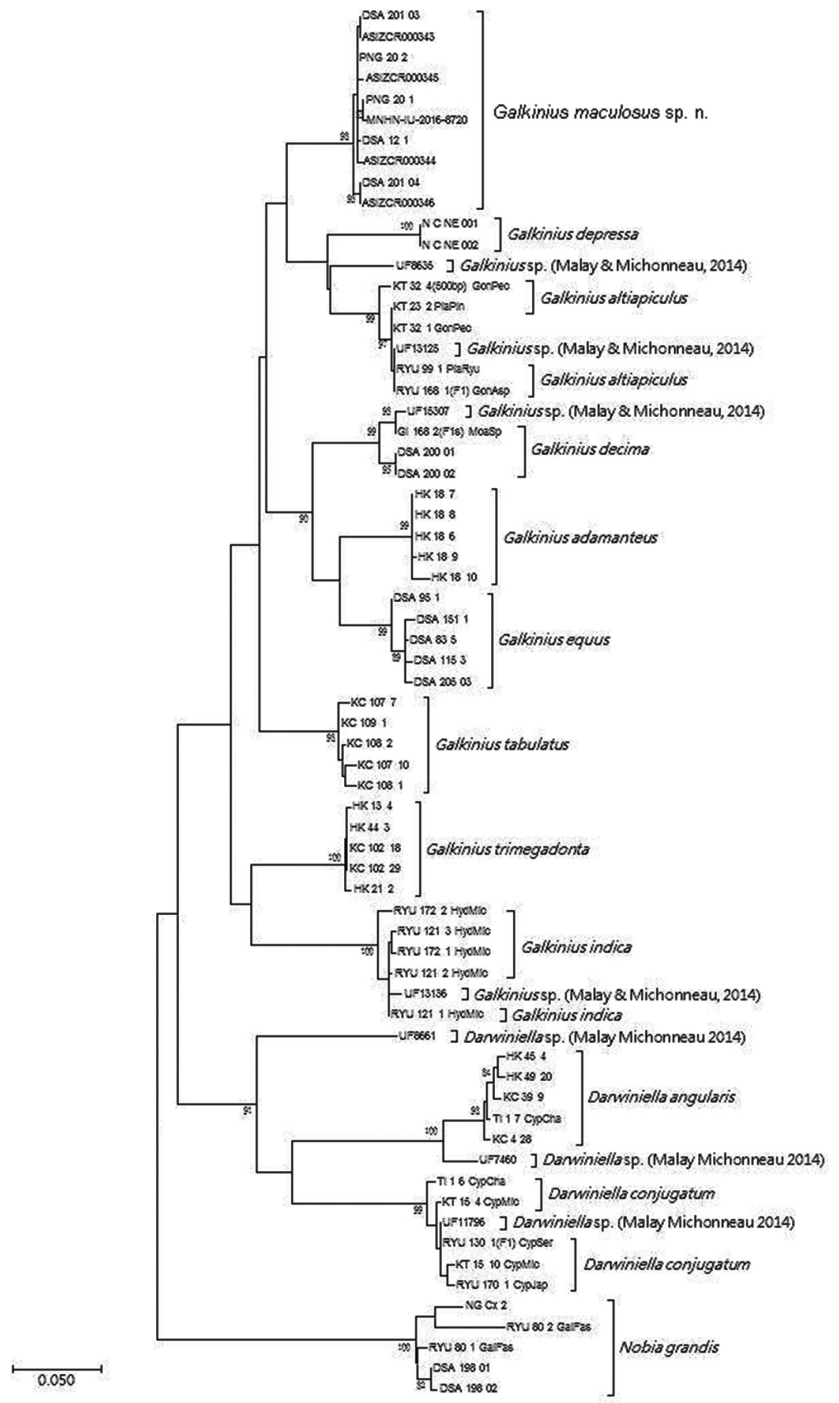
Maximum Likelihood (ML) method inferred genealogical relationships of *Darwiniella* and *Galkinius* specimens based on 624bp 12S and COI with *Nobia grandis* as the outgroup. Numbers above the major nodes are bootstrap values of 1000 replicates.

**Figure 11.**
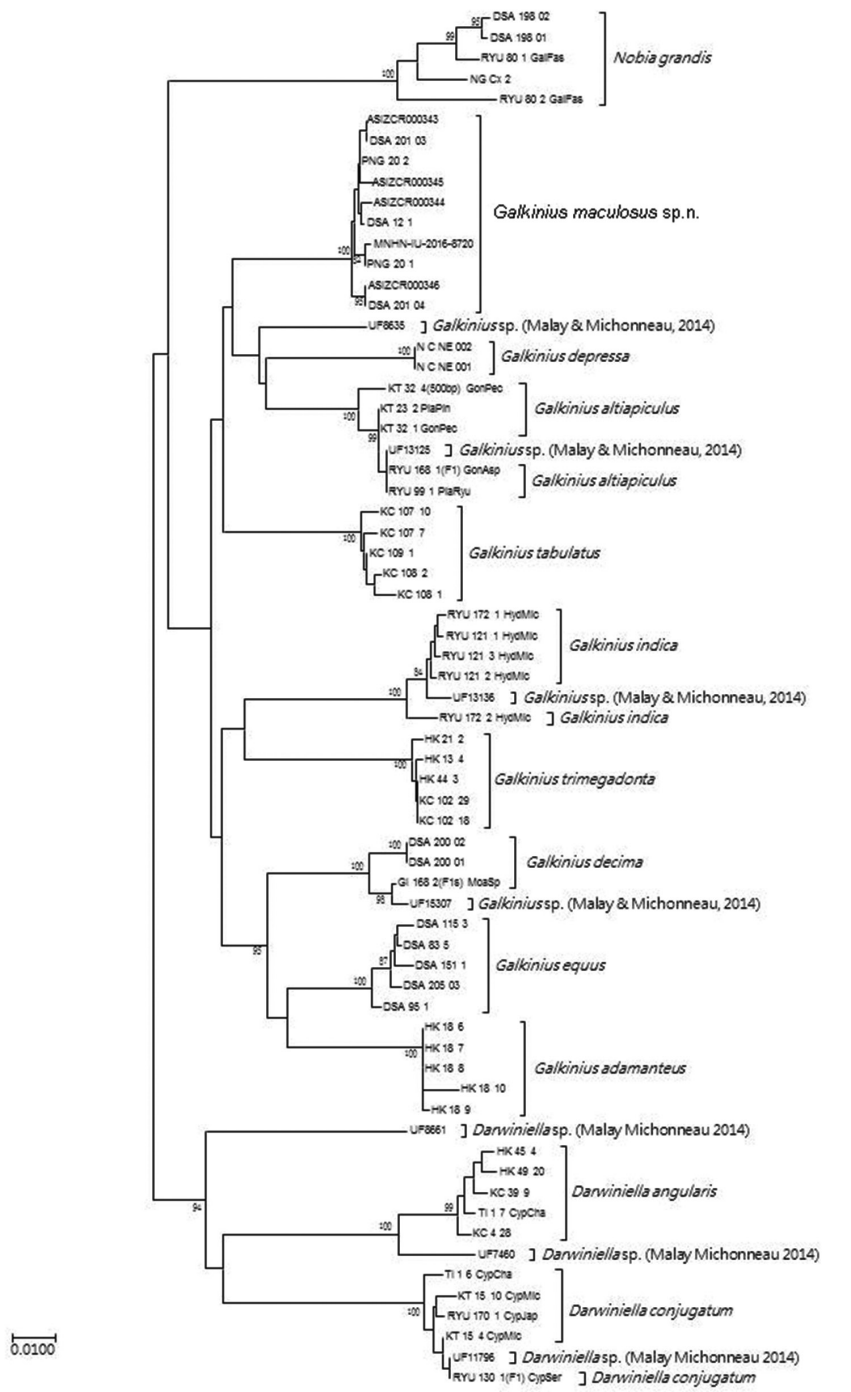
Neighbour-Joining (NJ) method inferred genealogical relationships of *Darwiniella* and *Galkinius* specimens based on 624bp 12S and COI with *Nobia grandis* as the outgroup. Numbers above the major nodes are bootstrap values of 1000 replicates.

After trimming and aligning the sequences, 624bp of 12S and COI rDNA were obtained from 23 *Darwiniella* specimens and 39 *Galkinius* specimens without indels, respectively (Fig. 10, 11, Table 1: sequence data). Evolutionary distances based on p-distance/T92-distance were 0.009/0.009, 0.008/0.008 and 0.005/0.005 within *D. angularis*, *D. conjugatum*, and *G. maculosus* sp. n., respectively, and 0.109/0.119 between *D. angularis* and *D. conjugatum*, 0.124/0.136 between *D. angularis* and *D. maculosus* sp. n., 0.112/0.122 between *G. maculosus* sp. n. and *D. conjugatum*. Sequence UF11796 (Malay and Michonneau 2014) was clustered in the *D. conjugatum* clade and with between group evolution distance p-distance/T92-distance equaled to 0.005/0.005 which indicated this sequence should be *D. conjugatum*. Other two sequences UF8661 and UF7460 did not include in any identified *Darwiniella* clades and the evolutionary distances based on p-distance/T92-distance were 0.099/0.107 between UF8661 and *D. conjugatum*, 0.036/0.037 between UF8661 and *D. angularis*, 0.116/0.126 between UF8661 and *G. maculosus* sp. n, 0.095/0.103 between UF7460 and *D. conjugatum*, 0102/0.110 between UF7460 and *D. angularis*, 0.102/0.111 between UF7460 and *G. maculosus* sp. n. Therefore, these two sequences may represent two additional undescribed *Darwiniella* species.

**Table 1.** Reporting table of ranking sequence reliability and accession numbers of GenBank submission.

Specimen catalog	Species name	Reliability ranking	Source materials	GenBank	
				12S	COI
ASIZCR000343	*Galkinius maculosus* sp. n.	1^st^	Holotype	KY575518	KY575512
ASIZCR000346	*Galkinius maculosus* sp. n.	2^nd^	Paratype	KY575514	KY575509
ASIZCR000344	*Galkinius maculosus* sp. n.	2^nd^	Paratype	KY575516	KY575510
ASIZCR000345	*Galkinius maculosus* sp. n.	2^nd^	Paratype	KY575517	KY575511
MNHN-IU-2016-8720	*Galkinius maculosus* sp. n.	2^nd^	Paratype	KY575515	KY575513
DSA_12_1	*Galkinius maculosus* sp. n.	4^th^	Non-type additional specimen	KY419721	KY419776
DSA_201_03	*Galkinius maculosus* sp. n.	4^th^	Non-type additional specimen	KY419722	KY419777
DSA_201_04	*Galkinius maculosus* sp. n.	4^th^	Non-type additional specimen	KY419723	KY419778
PNG_20_1	*Galkinius maculosus* sp. n.	4^th^	Non-type additional specimen	KY419724	KY419779
PNG_20_2	*Galkinius maculosus* sp. n.	4^th^	Non-type additional specimen	KY419725	KY419780
HK_45_4	*Darwiniella angularis*	4^th^	Non-type additional specimen	KY419711	KY419766
HK_49_20	*Darwiniella angularis*	4^th^	Non-type additional specimen	KY419712	KY419767
KC_4_28	*Darwiniella angularis*	4^th^	Non-type additional specimen	KY419713	KY419768
KC_39_9	*Darwiniella angularis*	4^th^	Non-type additional specimen	KY419714	KY419769
TI_1_7_CypCha	*Darwiniella angularis*	4^th^	Non-type additional specimen	KY419715	KY419770
KT_15_4_CypMi	*Darwiniella conjugatum*	4^th^	Non-type additional specimen	KY419716	KY419771
KT_15_10_CypMic	*Darwiniella conjugatum*	4^th^	Non-type additional specimen	KY419717	KY419772
RYU_130_1_CypSer	*Darwiniella conjugatum*	4^th^	Non-type additional specimen	KY419718	KY419773
RYU_170_1_CypJap	*Darwiniella conjugatum*	4^th^	Non-type additional specimen	KY419719	KY419774
TI_1_6_CypCha	*Darwiniella conjugatum*	4^rd^	Non-type additional specimen	KY419720	KY419775
HK_18_6	*Galkinius adamanteus*	4^th^	Non-type additional specimen	KY419726	KY419781
HK_18_7	*Galkinius adamanteus*	4^th^	Non-type additional specimen	KY419727	KY419782
HK_18_8	*Galkinius adamanteus*	4^th^	Non-type additional specimen	KY419728	KY419783
HK_18_9	*Galkinius adamanteus*	4^th^	Non-type additional specimen	KY419729	KY419784
HK_18_10	*Galkinius adamanteus*	4^th^	Non-type additional specimen	KY419730	KY419785
KT_23_2_PlaPin	*Galkinius altiapiculus*	4^th^	Non-type additional specimen	KY419731	KY419786
KT_32_1_GonPec	*Galkinius altiapiculus*	4^th^	Non-type additional specimen	KY419732	KY419787
KT_32_4_GonPec	*Galkinius altiapiculus*	4^th^	Non-type additional specimen	KY419733	KY419788
RYU_99_1_PlaRyu	*Galkinius altiapiculus*	4^th^	Non-type additional specimen	KY419734	KY419789
RYU_168_1_GonAsp	*Galkinius altiapiculus*	4^th^	Non-type additional specimen	KY419735	KY419790
DSA_200_01	*Galkinius decima*	4^th^	Non-type additional specimen	KY419736	KY419791
DSA_200_02	*Galkinius decima*	4^th^	Non-type additional specimen	KY419737	KY419792
GI_168_2_MoaSp	*Galkinius decima*	4^th^	Non-type additional specimen	KY419738	KY419793
N_C_NE_001	*Galkinius depressa*	4^th^	Non-type additional specimen	KY419739	KY419794
N_C_NE_002	*Galkinius depressa*	4^th^	Non-type additional specimen	KY419740	KY419795
DSA_83_5	*Galkinius equus*	4^th^	Non-type additional specimen	KY419741	KY419796
DSA_95_1	*Galkinius equus*	4^th^	Non-type additional specimen	KY419742	KY419797
DSA_115_3	*Galkinius equus*	4^th^	Non-type additional specimen	KY419743	KY419798
DSA_151_1	*Galkinius equus*	4^th^	Non-type additional specimen	KY419744	KY419799
DSA_205_03	*Galkinius equus*	4^th^	Non-type additional specimen	KY419745	KY419800
RYU_121_1_HydMic	*Galkinius indica*	4^th^	Non-type additional specimen	KY419746	KY419801
RYU_121_2_HydMic	*Galkinius indica*	4^th^	Non-type additional specimen	KY419747	KY419802
RYU_121_3_HydMic	*Galkinius indica*	4^th^	Non-type additional specimen	KY419748	KY419803
RYU_172_1_HydMic	*Galkinius indica*	4^th^	Non-type additional specimen	KY419749	KY419804
RYU_172_2_HydMic	*Galkinius indica*	4^th^	Non-type additional specimen	KY419750	KY419805
KC_107_7	*Galkinius tabulates*	4^th^	Non-type additional specimen	KY419751	KY419806
KC_107_10	*Galkinius tabulates*	4^th^	Non-type additional specimen	KY419752	KY419807
KC_108_1	*Galkinius tabulatus*	4^th^	Non-type additional specimen	KY419753	KY419808
KC_108_2	*Galkinius tabulatus*	4^th^	Non-type additional specimen	KY419754	KY419809
KC_109_1	*Galkinius tabulatus*	4^th^	Non-type additional specimen	KY419755	KY419810
HK_13_4	*Galkinius trimegadonta*	4^th^	Non-type additional specimen	KY419756	KY419811
HK_21_2	*Galkinius trimegadonta*	4^th^	Non-type additional specimen	KY419757	KY419812
HK_44_3	*Galkinius trimegadonta*	4^th^	Non-type additional specimen	KY419758	KY419813
KC_102_18	*Galkinius trimegadonta*	4^th^	Non-type additional specimen	KY419759	KY419814
KC_102_29	*Galkinius trimegadonta*	4^th^	Non-type additional specimen	KY419760	KY419815
DSA_198_01	*Nobia grandis*	4^th^	Non-type additional specimen	KY419761	KY419816
DSA_198_02	*Nobia grandis*	4^th^	Non-type additional specimen	KY419762	KY419817
NG_Cx_2	*Nobia grandis*	4^th^	Non-type additional specimen	KY419763	KY419818
RYU_80_1_GalFas	*Nobia grandis*	4^th^	Non-type additional specimen	KY419764	KY419819
RYU_80_2_GalFas	*Nobia grandis*	4^th^	Non-type additional specimen	KY419765	KY419820

All the *Darwiniella* and *Galkinius* specimens can be divided into two clades, one contains two *Darwiniella* species (*D. angularis* and *D. conjugatum*) while the remaining species (*G. maculosus* sp. n. and all the *Galkinius* species) construct the second clade. All the bootstrap values of the nodes which separate these two clades are above 80 and therefore these nodes are well supported.

## Discussion

*Galkinius maculosus* sp. n. has shared similarities between *Galkinius* and *Darwiniella*. There are two possible genera for *Galkinius maculosus* sp. n. Based on the fused shell and opercular plates, *Galkinius maculosus* sp. n. can be placed under *Darwiniella*. Subsequently, the molecular phylogenetic pattern of *Dawiniella* will become diphyletic, with *D. conjugatum* and *D. angularis* in one molecular clade, and *Galkinius maculosus* sp. n. (if identified as *Darwiniella*) will be located in the other molecular clade with *Galkinius* species together. Identification of *Galkinius maculosus* sp. n. under the genus *Darwiniella*, based only on its fused shell character, probably trumps in characters of somatic body, tergum shape and molecular data.

**Figure 12.**
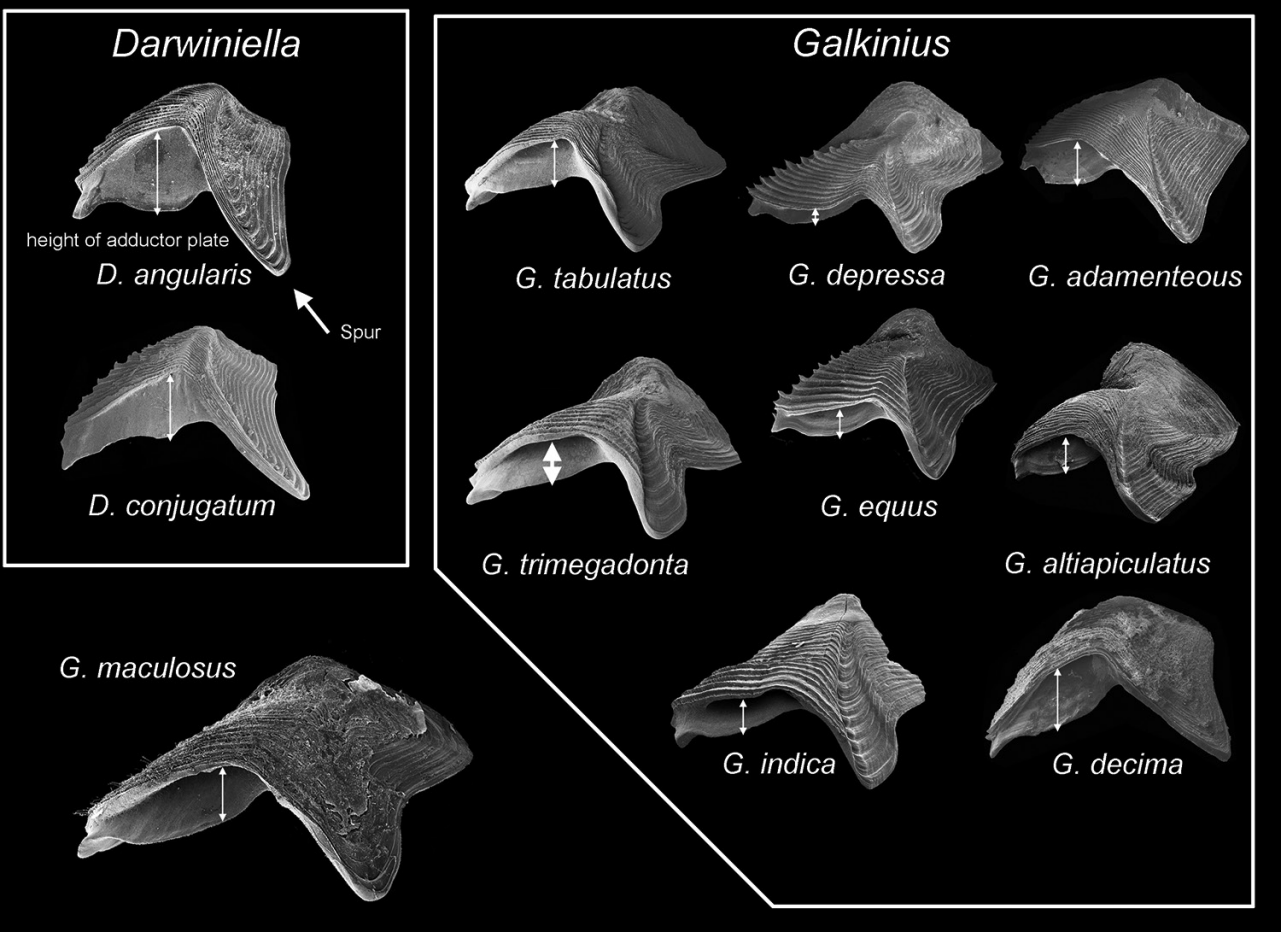
Comparisons of opercular plates (fused scutum and tergum) of *Galkinius maculosus* sp. n. among species in *Darwiniella* and *Galkinius*. Note the height of adductor plate (indicated by double arrows) is much greater in *Darwiniella* than *Galkinius* species. The spur of tergum (indicated by single arrow) is sharper in *Darwiniella* than *Galkinius*. The opercular plate of *G. maculosus* sp. n. is closer to species in *Galkinius*.

Apart from the character of fused shell, there are many morphological characters of *Galkinius maculosus* sp. n. which fit well to *Galkinius* rather than *Darwiniella*. The shape of the opercular plates, especially the wide spur in the tergum of *Galkinius maculosus* sp. n., is similar to species of *Galkinius* (Fig. 12; also see Chan et al. 2013, Simon-Blecher et al. 2016). The adductor plate of *Galkinius maculosus* sp. n. is narrow, which is similar to other *Galkinius* species, rather than the wide adductor plate in *Darwiniella* (Fig. 12). The aperture frill, maxilla, mandibular palp, and cirrus of *Galkinius maculosus* sp. n. are spotted, similar to those of *Galkinius*, in contrast to those of species of *Darwiniella* which have very few spots. The size of the *Galkinius maculosus* sp. n. is comparable to *Galkinius* (see Chan et al. 2013) and much larger than *Darwiniella* (see Chen et al. 2012). Adults of *Galkinius maculosus* sp. n. are approximately twice as large as *D. angularis* and one and a half times larger than *D. conjugatum*. Based on the morphological similarities of *Galkinius maculosus* sp. n. to *Galkinius*, this species is classified under *Galkinius* and, in this case, the monophyly of *Darwiniella* and *Galkinius* in the molecular phylogeny tree is preserved.

The sequences divergence of the two *Darwiniella* species (UF8661 and UF7460) from Malay and Michonneau (2014) clustering into the clades with the *Darwiniella* species further supports the monophyly of *Darwiniella*. These two *Darwiniella* sequences from Malay and Michonneau (2014) were collected in the Oman and the Philippines, indicating that there is further diversity within *Darwiniella* waiting to be explored in the Pacific and Indian oceans.

## Supplementary Material

XML Treatment for Galkinius

XML Treatment for Galkinius maculosus
